# Why do young adults gamble online? A qualitative study of motivations to transition from social casino games to online gambling

**DOI:** 10.1186/s40405-017-0025-4

**Published:** 2017-08-22

**Authors:** Hyoun S. Kim, Michael J. A. Wohl, Rina Gupta, Jeffrey L. Derevensky

**Affiliations:** 1 University of Calgary, Calgary, Canada; 2 Carleton University, Ottawa, Canada; 3 Queens University, Kingston, Canada; 4 McGill University, Montreal, Canada

**Keywords:** Online gambling, Adolescent gambling, Social media, Social casino games, Facebook, Focus groups, Qualitative research

## Abstract

The present research examined the mechanisms of initiating online gambling among young adults. Of particular interest was whether social casino gaming was noted as part of young adults’ experience with online gambling. This is because there is growing concern that social casino gaming may be a ‘gateway’ to online gambling. Three focus groups (*N* = 21) were conducted with young adult online gamblers from two large Canadian Universities. Participants noted the role of peer influence as well as incentives (e.g., sign up bonuses) as important factors that motivated them to start engaging in online gambling. Participants also noted a link between social casino games and online gambling. Specifically, several young adults reported migrating to online gambling within a relatively short period after engaging with social casino games. Potential mechanisms that may lead to the migration from social casino games to online gambling included the role of advertisements and the inflated pay out rates on these free to play gambling like games. The results suggest initiatives to prevent the development of disordered gambling should understand the potential of social casino gaming to act as a gateway to online gambling, especially amongst this vulnerable population.

Over the past decade, the use of computers and the Internet has significantly altered the gambling landscape. The gambling industry is no longer bound by brick and mortar gambling venues (e.g., casinos, racetracks). Today, access to gambling activities can be achieved with a few keystrokes on a computer. One point of access that has gained increased attention from researchers in the field of gambling studies is social media sites such as *Facebook* (Wohl et al. 2017). In part, this increased attention is because social media sites have become a popular platform for people to access online gambling venues via hyperlinks embedded in advertisements (Abarbanel et al. 2016). Social media sites also allow users to engage in free-to-play simulated gambling games through applications. These free-to-play simulated gambling games have become referred to as social casino games (Gainsbury et al. 2014). There is evidence to suggest, however, that social casino game play may act as a ‘gateway’ to gambling for real money (for a review see Wohl et al. 2017).

The current research took a qualitative approach to assess young adult online gamblers experiences with online gambling to determine the process and mechanisms that may lead young adults to gamble online, including the role of social casino games. In other words, the present research aimed to examine the motivations for gambling online, including transitioning from social casino games to online gambling. A focus was placed on young adults’ experience with online gambling due to their propensity to gamble online (McBride and Derevensky 2009), play social casino games (Derevensky and Gainsbury 2016), as well as their elevated rates of disordered gambling (Welte et al. 2011). Further, social casino games were the focus as there is a current need to understand the issues regarding the gaming-social media crossover.

## Online gambling and social casino gambling among young adults

The Internet has changed the way people engage in many activities, including gambling. Online gambling (compared with land-based gambling) provides players with ease of access, 24/7 accessibility, and confidentiality—all within the comfort of a person’s home. This ease of access has been flagged as a potential concern among researchers, regulators, and policy makers alike (Gainsbury 2015; Gainsbury and Wood 2011; Räsänen et al. 2013). Specifically, online gambling is often framed as a ‘risky’ form of gambling that may heighten the risk of developing a gambling disorder (Gainsbury et al. 2015b; Griffiths et al. 2009; McBride and Derevensky 2009; Olason et al. 2011; Wood et al. 2007). In this light, it may be informative to examine factors that propel young adults to gamble online, including the link between social casino gaming and online gambling. This is because there is increasing evidence of the role played by social casino games in precipitating online gambling (Wohl et al. 2017) and young adults are increasingly exposed to social casino games (Kim et al. 2016).

Social casino games are an immensely popular form of entertainment, with millions of users playing in any given day (Derevensky and Gainsbury 2016; Martin 2014). One reason for their popularity may be their ubiquity on social network sites like *Facebook*, which provide ample opportunities to play social casino games via embedded apps (Gainsbury et al. 2014). Moreover, social casino games are among the most heavily advertised products on social network sites and convey the activity (i.e., gambling) as positive and glamorous (Gainsbury et al. 2015a). These advertisements appear to have a significant influence on engagement with social casino games (SuperData 2016). It should be noted that some social casino games are now owned by online gambling operators who advertise their online gambling site within the social casino game, thus easing migration from social casino gaming to online gambling (Schneider 2012).

There is now converging evidence that suggests social casino gamers migrate to online gambling (Gainsbury et al. 2016; Kim et al. 2015). Furthermore, amongst people who engage in both gambling and social casino gaming, social casino games directly increase future gambling behaviors (Gainsbury et al. 2016, 2017; Hollingshead et al. 2016). Social casino games are also popular among adolescents and young adults. In a large Canadian survey of over 10,000 students, roughly 9% reported having played social casino games (Elton-Marshall et al. 2016). In addition, a recent longitudinal study in a large sample of adolescents found that social casino games significantly predicted the transition to real money gambling (Dussault et al., in press). Providing further support for the popularity of social casino games, in focus groups with university students who were social media users, all participants reported being aware of the ample opportunities to play social casino games on Facebook, thus speaking to the increased exposure of these games on social networking sites (Kim et al. 2016).

### Motivations for transitioning to online gambling from social casino gaming

Social casino games are popular among adolescents and young adults and may influence the transition to online gambling. Yet, researchers have paid little attention to potential processes or mechanisms that influences the transition to online gambling amongst this cohort, including the role played by social casino games. With that said, Hollingshead et al. (2016) argued that the motivations for playing social casino games likely mimic those of online gambling, including for excitement, to relieve boredom, and social motivations. In addition, they reported that some social casino gamers are motivated to engage in these games to hone their skills before playing for real money on online gambling sites. In line with Hollingshead et al. (2016) and King and Delfabbro (2016) proposed a framework for understanding factors that may increase or decrease the link between social casino gaming and online gambling among adolescents. Specifically, in their two pathways model, they identify both protective (e.g., early losses, awareness of risks, boredom) and risk factors (e.g., peer pressure, early big wins, greater confidence of winning) that may lead adolescents who are exposed to social casino games to either be disinterested in gambling or to increase future gambling behaviors.

The present research sought to add to the growing literature on the potential link between social casino gaming and online gambling. To do so, focus groups with young adult online gamblers were conducted to explore their motivations for gambling online, including the potential role social casino games played in initiating or facilitating online gambling behaviors. Focus groups provide a compromise between obtaining personal experiences without having to interview people individually, while also having a group environment where other people’s experience stimulate the recall and views of others. In this light, focus groups are an effective method of obtaining a variety of detailed information in an exploratory way.

## Methods

### Participants

Twenty-one young adults (18 males, 3 females) were recruited from two large Canadian Universities to participate in one of three focus groups described as being about young adults’ experience with online gambling. Specifically, the study was advertised as a focus group for people who gambling online. It was explained that we were interested in online gamblers’ “opinions and experiences regarding online gambling”.

The inclusion criteria were as follows: college students aged 18–24 years who reported gambling online at least twice per month. The method of recruitment occurred in two ways. First, all incoming first year students at one of the large Canadian universities complete a short survey screening for disordered gambling. Embedded in that questionnaire were items that assessed online gambling. This allowed us to recruit participants who met the inclusion criteria for the focus groups. Only those who consented to be recruited for future studies were contacted. The second method of recruitment consisted of visiting large classrooms and advertising the study at both universities.

While every effort was made to recruit an equal number of male and female online gamblers we were unable to do so despite our best efforts. Moreover, seven individuals who had initially agreed to participate in the study subsequently notified the research team before the group meeting that they could not participate for logistical reasons (i.e., work and school commitments, unexpected appointments). Participants in the first group were compensated $20 for their time and those in the remaining two groups were provided with $40 (the increased compensation was used as an incentive to attract a greater number of participants and was cleared by the authors’ Research Ethics Board). Additionally, participants were provided food and beverages throughout the course of the discussions that ensued.

### Procedure and materials

All participants were provided with a description of the study objectives and were asked to read and sign an informed consent prior to participating in the current research. Participants were informed they were free to terminate participation at any time without penalty. Thereafter, participants were asked to complete a short background questionnaire, which included demographic information (gender, age), frequency of gambling, and how knowledgeable they believe themselves to be on the topic of online gambling.

A series of open-ended questions were asked of the group as part of a larger project assessing online gambling among young adults. For the present research, two open-ended questions were of importance. The first examined general factors that lead young adults to gamble online, “I’d like to gain a better understanding of the things that lead to online gambling in the first place. Based on what you know, what are the factors, the events, or the influences that result in a young person deciding to bet money on gambling activities online?” The second assessed the social casino game-online gambling link including the potential mechanisms, “You know that social media sites have gambling-type games such as Texas-Hold’em or Sloto-mania. In your opinion, do you think experience with these games leads a person to seek online gambling sites? In other words, do these types of games serve as a form of initiation to gambling online with real money?”

A licensed clinical psychologist trained in conducting focus groups led the discussions accompanied by two note-takers. Each group was approximately 60–75 min in duration and discussions were conducted at two Canadian universities. Two recording devices recorded the focus group to ensure no loss of data. Upon the completion of the focus groups, the discussions were subsequently transcribed by a professional coder and coded by two independent reviewers. The initial categories generated by the data were highly consistent between the two raters with regards to general themes and number of categories. The data was reviewed two additional times to arrive at a consensus when disagreements between raters were noted. Categorical names were arrived through consensus after discussion between raters. NVivo 10 qualitative research software for qualitative analyses was used to organize and quantify the data.

## Results

With respect to frequency of online gambling, 52% of individuals indicated gambling less than once per week, while 48% indicated gambling at least once per week or several times per week. Seventy-six percent of individuals indicated gambling more frequently and/or for longer periods of time than intended (61.9% occasionally; 14.4% often). Participants were asked to indicate on a 7-point Likert scale how knowledgeable they perceived themselves to be on the topic of online gambling. The overall mean score was 4.38. The majority of the sample (85.7%) indicated that they tend to play on one or two online gambling sites, whereas 14.3% stated they like to experiment with different sites. Importantly, more than half (62%) of the participants revealed playing social casino games (e.g., *Texas Hold’em*) on Facebook or on other platforms. Of the participants (*n* = 3) who spontaneously reported having transitioned from playing for fun to online gambling, they did so in relatively short period of time. One participant reported transitioning after only two weeks, while another stated having moved to real money gambling after a couple of months.

### General factors leading to online gambling

Several themes emerged in regards to the factors that led the emerging adults to online gambling. For example, some of the emerging adults in the focus groups stated that friends played an important role in their initial participation to online gambling. Specifically, several participants reported having first learned to gamble with friends and thereafter transitioning to online gambling as their friends were not always available.

From my personal experience for example, I started gambling online with poker because I started playing poker with friends, and that is how I got to gambling online… with friends they did not always have the time [to play poker].

Gambling online was just easier – with friends they did not always have time.

Another theme that was noted in the precipitation of online gambling was the incentives (e.g., sign up bonuses) offered by online gambling. The young adult online gamblers noted that the first time they gambled online was when they were offered bonuses and free credits. Indeed, the participants agreed that the bonuses were an important incentive in moving to online gambling.

The bonuses actually attract us to them. You don’t get that at the casino.

For me the first incentive was they offered us 10 lb… so I got the 10 lb and then started betting real money

### Motivations from transitioning from social casino games to online gambling

Texas Hold’em with free chips, that’s how I started. A general progression starts with these Facebook entertainment games which are purely for fun and some people take it to the next level where it’s for fun and money, that’s where we are now - most of us and then some people will take it eventually to the next level where the fun has disappeared and they are just doing it for the money.

Social casino games were noted as a potential factor that influenced the initiation of online gambling among young adults. In fact, whilst the moderator had intentions to bring up social casino games as a topic, in all three focus groups, the young adult online gamblers spontaneously brought up social casino games. These results indicate that social casino games are a salient aspect of young adult online gamblers’ experiences. Not surprisingly, the young adult online gamblers mentioned the constant advertisements as a potential factor that may lead social casino gamers to online gambling. Specifically, the frequent nature of the advertisements that provided social media users with an opportunity was brought up by several focus group members, with few young adult online gamblers mentioned the role of advertisement in the transition to online gambling.

I’d argue that you are just sort of lured into playing more through back link advertising where you will have all these ads like partypoker.com keep coming back at you even when you are on other sites…

… and obviously the companies [social media] give out the information on things that you are doing like all the games and poker, even though it’s not for money. Your side bar has all advertisements that are personalized to you so for me I see a lot of gambling, sports, apparel stuff and stuff like that is all on my side bar.

When I started, it was Facebook. Randomly the opportunity comes up with ads. I was stressed so I went to the online casino from Facebook. Every day, every day, the online casino sends you notifications…

The young adult online gamblers also noted a link between social casino games and online gambling, with several participants stating they transitioned to online gambling after playing for free on Facebook. One potential implication is the inflated payout rate offered by social casino games. The focus group members noted they win more frequently on social casino games, which provides them a sense of hope that they would be winning money had they been gambling for real. There was a general sense of needing to be “smart” and “savvy” to not fall prey to the tactics of online casinos and social media sites.

Once you play for fun, they sort of get people into the gambling, you think ok, this would be great if it were real money, so you try. That’s the way the websites make you go through that road.

They want you to win… if you are winning on Facebook and then you see [an advertisement] on the side to go online to play at party poker you will think if I can do this for free I can do this for real and then you go to do it for real and the next thing you know you are down $150 when you were getting Blackjack with the other one [social casino site].

There was a consensus that social casino games provided an excellent learning opportunity. Specifically, social casino games allow people to learn rules, procedures, and strategies to gamble.

So regardless of whether it is Facebook or just the practice sites on the online casinos, it’s a natural progression to start from social casino games: train, learn… then you realize you are not learning enough because people are not taking the game seriously, and then you move onto paying.

I don’t know those procedures so I don’t play (in casinos). But online who is going to yell at you online? So like you can just practice online and you can play lower [limit] tables. Basically you can practice online without other people yelling at you.

Participants also noted that after playing for free, they transitioned to online in part as most players who play for free do not play the game the ‘right way’

The difference between a table with real money and a table with fake money, the people with fake money, they don’t do the moves they usually do with their real money. You just mess around, you don’t really care “Oh I’m all in” – it’s like you don’t care. But at the real tables everyone plays the way they want to play. You get to learn a lot when you play.

I started playing online and when I played online without money I realized this was not really like anywhere close to the situation you would be in at a real table cause you don’t have any money on it, so I decided to start gambling with money.

However, not everyone perceived a link between social casino games and online gambling. These individuals explained that the interfaces of the games were so different (social media being much less sophisticated) that people who are attracted to one would likely not be attracted to the other.

I don’t think it’s as dangerous as people make it to be. If I want to switch from gambling on Facebook to a real site I just go to Google and type in poker and have it [online site].

You start playing poker with your friends and like you move from that step onto other things. I don’t think you go from Facebook to gambling. I don’t see that as a gateway at all.

## Discussion

In today’s technological world, young adults are exposed to a plethora of opportunities to engage in gambling activities, including simulated gambling games on social media sites. For some young adults, exposure to gambling and gambling-like activities may result in the over-involvement of gambling. In three focus groups, motivations that influenced young adults to engage in online gambling were explored. The participants noted several factors that motivated them to engage in online gambling: including suggestions from friends, the ease and accessibility of online gambling (compared to land-based venues), and incentives offered by the online gambling operators (e.g., $10 in free play).

The results of our present research may have important implications for the progression and maintenance of online gambling among young adults. First, several participants reported having been drawn to online gambling by bonuses offered by the gambling operators. Whilst incentives may help attract new customers, it should be noted that they may not be creating frequent customers. Indeed, free-play offers (e.g., bonus offers) bring customers into a gambling venue, but fail to generate significant increases in volume of play (Lucas et al. 2005). Having said that, given that online gambling is often framed as a risky form of gambling, in part due to the increased accessibility, whether operators should be allowed to offer incentives, especially amongst vulnerable population may be an important question which policy makers should address.

In addition to general factors that may motivate young adults to engage in online gambling, potential mechanisms for the social casino games-online gambling link were explored. One potential mechanism noted by the participants that may lead to the migration of online gambling from social casino games involves the use of advertisements by the online gambling operators. Specifically, it was noted that gambling operators sometimes use social casino games to advertise gambling activities without legal restrictions because it is a game. Indeed, as social casino games are not technically gambling activities, there is no regulation in regards to advertisement, prompting some to suggest that advertisements for social casino games be held to the same standard as gambling (Gainsbury et al. 2014). It has been suggested that these advertisements are more likely to appear to young adults and adolescents (Abarbanel et al. 2016). Further, advertisements for gambling (including social casino games) are frequent on social media sites and portray the positive aspects of gambling without any of the potential dangers (Gainsbury et al. 2016). Some of the participants in the focus groups reported moving from social casino games to real money gambling due to the constant advertisements of online casinos. As young adults may be more likely to be influenced by advertisements (Derevensky et al. 2010), some researchers have suggested that advertisements for social casino games be held to the same standard as gambling (Derevensky and Gainsbury 2016). Our results seem to provide support for this suggestion.

A second mechanism by which players migrated from social casino games to online gambling was via the inflated payout rates on social casino games. Note this mechanism was also identified in the two pathways model proposed by King and Delfabbro (2016). Specifically, participants felt an increased confidence in winning should they have engaged in real-money gambling. Further, several participants stated that their frequent wins on social casino games propelled them to try engaging in online gambling. This is in line with previous research, which found that a portion of casino gamers play these games to build up their ‘skill’ before migrating to gambling in land-based or online gambling venues (see Kim et al. 2016). However, the inflated payout rates may give players an inflated belief in the skill, and, of course, there is no skill if the game of choice is one of pure-chance, like a slot machine. In fact, social casino game outcomes are not based on random odds and mathematics, but are rather designed to enhance player enjoyment (Wohl et al. 2017). Because of this, the social casino gamer wins more than he loses (Sévigny et al. 2005), which in turn, may falsely increase their confidence in winning, as proposed by King and Delfabbro (2016). Providing further support that frequent wins and perception of skills as a process by which social casino games to lead to online gambling, Hollingshead et al. (2016) showed that playing social casino games for skill purposes have been linked to problematic gambling behaviors. In this light, it would behoove regulators to enforce payout rates that are similar to gambling activities, or at very least mandate social casino gaming operators to inform players of that social casino games are not based on random odds as their gambling counterparts.

According to Blaszczynski and Nower’s (2002) pathways model of problem and pathological gambling, there are three distinct subgroups of gamblers, each with different pathways that manifest in problem gambling behaviors. In the model, the starting point is ecological factors, which include increased availability and accessibility. In this way, social casino games may influence the development of problem gambling among young adults by providing ease of access and increased availability. Indeed, one of the concerns of social casino games is that although they purport to have age verifications, a UK study found that 300,000 youths aged 11–16 reported having engaged in free online gambling games in the past week (Parke et al. 2013). Furthermore, it is plausible that if social casino games lead to the development of problem gambling, it does through Pathway 1, the behaviourally conditioned gambler. This pathway includes cognitive mechanisms such as irrational beliefs and illusion of control, which may manifest due to the inflated payout rates on social casino games. That said, this is an assertion and would be in need of empirical support.

### Limitations

Some limitations of the current study should be noted. First, we did not recruit a sufficient number of female online gamblers to ascertain different trends and cognitions that may be gender-specific. That said, studies have consistently found that online gamblers tend to be young males (Griffiths et al. 2009; for a review see Gainsbury 2015). Thus, we have confidence that the observed results maintain ecological validity. Secondly, the findings of the current project are not intended to be reflective of the college population as a whole. Rather, the findings are qualitative in nature and should be used to guide future research initiatives. Lastly, we recruited online gamblers to participant in the focus groups, rather than social casino gamers. Thus, the current study cannot speak to social casino games being a deterrent to online gambling (e.g., knowing you can’t win).

## Conclusion

The Internet has drastically shaped the way in which people engage with the world, including with gambling activities. Furthermore, social networking sites have become a fabric of the modern day world. While the Internet and specifically social networking sites are a great medium to stay connected with loved ones, they have increasingly become an avenue to engage in gambling activities, including simulated forms of gambling (i.e., social casino games). The present research explored the motivations that push young adults to engage in online gambling, including the role of social casino games. Further research and attention is needed in this domain to mitigate the potential migration from gaming to gambling, specifically amongst those most vulnerable.
